# Functionalized Soybean Oil- and Vanillin-Based Dual Cure Photopolymerizable System for Light-Based 3D Structuring

**DOI:** 10.3390/polym14245361

**Published:** 2022-12-08

**Authors:** Vilte Sereikaite, Aukse Navaruckiene, Justinas Jaras, Edvinas Skliutas, Dimitra Ladika, David Gray, Mangirdas Malinauskas, Vaidas Talacka, Jolita Ostrauskaite

**Affiliations:** 1 Department of Polymer Chemistry and Technology, Kaunas University of Technology, Radvilenu Rd. 19, LT-50254 Kaunas, Lithuania; 2 Laser Research Center, Faculty of Physics, Vilnius University, Sauletekis Ave. 10, LT-10223 Vilnius, Lithuania; 3 Institute of Electronic Structure and Laser, Foundation for Research and Technology-Hellas, 70013 Heraklion, Greece; 4 AmeraLabs, Kęstučio Str. 6A, LT-44320 Kaunas, Lithuania

**Keywords:** vanillin dimethacrylate, acrylated epoxidized soybean oil, photocross-linking, dual-curing, stereolithography, two-beam initiation threshold, multiphoton polymerization

## Abstract

A novel dual cure photopolymerizable system was developed by combining two plant-derived acrylic monomers, acrylated epoxidized soybean oil and vanillin dimethacrylate, as well as the thiol monomer pentaerythritol tetrakis (3-mercaptopropionate). Carefully selected resin composition allowed the researchers to overcome earlier stability/premature polymerization problems and to obtain stable (up to six months at 4 °C) and selectively-polymerizable resin. The resin demonstrated rapid photocuring without an induction period and reached a rigidity of 317.66 MPa, which was more than 20 times higher than that of the other vanillin-based polymers. Improved mechanical properties and thermal stability of the resulting cross-linked photopolymer were obtained compared to similar homo- and copolymers: Young’s modulus reached 4753 MPa, the compression modulus reached 1634 MPa, and the temperature of 10% weight loss was 373 °C. The developed photocurable system was successfully applied in stereolithography and characterized with femtosecond pulsed two-beam initiation threshold measurement for the first time. The polymerization threshold of the investigated polymer was determined to be controlled by the sample temperature, making the footprint of the workstations cheaper, faster, and more reliable.

## 1. Introduction

Considering recent challenges related to climate change and the state of the environment, it is particularly important to develop new materials that combine specific technical and optional smart properties with sustainability and applicability for high-tech manufacturing. Light-based 3D structuring is a unique contactless fabrication method that offers material processing precision, flexibility, and the rapid manufacturing of mechanical, medical, and optical components and devices [1]. Real-time synchronization of fast beam deflection and precise sample positioning can be implemented for state-of-the-art mesoscale ultrafast laser 3D structuring, namely, the production of submicrometer precision functional prototypes of practically-applicable millimeter scale dimensions [2]. This breakthrough technological advancement makes laser 3D printing suitable and appealing for both scientific research and industrial scale additive manufacturing. For such a technology, the replacement of petroleum-based materials by plant-derived materials would provide immediate ecological and long-term economic benefits.

Although the use of biorenewable polymers is increasing in various applications, the main challenge remains, to develop biobased polymer analogues with the same or better technical properties (reactivity, stability, durability, etc.) as petroleum-based polymers [3]. Plant oils and plant phenolics are great biorenewable sources of starting materials for polymer synthesis [4,5]. Acrylated epoxidized soybean oil (AESO) with a high number of functional groups is produced industrially and is widely used in foams, adhesives, coatings, and even in light-based 3D printing [6,7], including multiple scales and exposure sources [8]. However, the use of pure AESO leads to poor mechanical and thermal properties of the resulting products [9]. This issue can be solved by adding various comonomers with rigid structure to the resin composition [10]. In this study, vanillin dimethacrylate was chosen as a biobased comonomer because of its aromatic structure and high reactivity. Vanillin is one of the few biobased aromatic compounds that are industrially available [11]. Furthermore, vanillin-based polymers demonstrate good mechanical and thermal properties, as well as extraordinary antimicrobial activity similar to that of chitosan-based polymers [12,13]. In this study, the best features of both biobased monomers, AESO and vanillin dimethacrylate, were combined in the dual-curing photopolymerizable system. Although dual cure is a very promising technique for controlling the structure and properties of 3D-printed polymeric objects, there are only a few examples of the usage of AESO [14] or vanillin derivatives [15] in dual cure systems.

Dual cure is a technique that combines two simultaneous or sequential curing reactions of the same or a different energy source [16]. As a result, interpenetrating or semi-interpenetrating polymer networks with unique properties are formed [17]. Acrylic monomers are widely used in dual-curing systems [18] and the resulting polymers with numerous industrial applications, such as adhesives, coatings, and biomaterials, can be synthesized by carefully selecting the acrylate monomer [19]. The most common comonomers for acrylates are thiols. They react with acrylates in two different ways, the thiol-acrylate Michael addition and the radical-mediated thiol-acrylate reaction. Both mechanisms are appropriate for the formation of dual-curing systems [18].

In this study, radical-mediated thiol-acrylate photopolymerization and acrylate homopolymerization were used to compose a novel dual-curing system. Two different biobased monomers, acrylated epoxidized soybean oil and vanillin dimethacrylate, were used with pentaerythritol tetrakis(3-mercaptopropionate) for the first time. Diphenyl(2,4,6-trimethylbenzoyl) phosphine oxide was selected as the photoinitiator due to its photobleaching effect and its ability to cure deep layers of resin [20]. The developed photopolymerizable system was stable and well-suited for the light-based 3D structuring of complex shape objects with excellent mechanical properties and thermal stability. Furthermore, the order of effective nonlinear absorption (*n_eff_*) in photoresists is a key element in the development of materials with improved sensitivity for additive manufacturing based on multiphoton absorption polymerization [21]. Z-can, intensity-scan, or a pump and probe technique can be used to determine the *n_eff_*. However, it characterizes material properties rather than the order of undergoing the polymerization reaction process. Other indirect methods such as non-linear fluorescence excitation or thermal lensing can be implemented for this task; however, they do not measure the *n_eff_* directly. Line-width [22] and exposure-time duration (ETM) [23] techniques allow for the determination of the *n_eff_* from the polymerized structures; however, both suffer from the required additional steps such as sample posttreatment and scanning electron microscopy, which are time consuming for systematic measurements. The pros and cons of the aforementioned approaches are well-discussed in a review paper by N. Liaros and J. T. Fourkas [24]. A quick, reliable, and, most importantly, in situ method is highlighted as a two-beam initiation threshold (2-BIT) [25]. Also, the method includes normalization of the power of each beam to the polymerization threshold power to correct the differences in beam size, pulse length, or focal volume. While other scientific groups are looking for ways to simplify two-photon polymerization techniques via laser systems [26,27], light projection conditions [28], or photoinitiation agents [29,30], in this work, a method of using impact of dual cure is presented. The 2-BIT experiment was performed for the *in situ* measurement of nonlinear material excitation mechanisms and, in principle, validated its suitability for laser 3D nanolithography, while revealing important peculiarities of nontrivial material behaviour depending on sample handling conditions.

## 2. Materials and Methods

### 2.1. Materials

**Figure 1:**
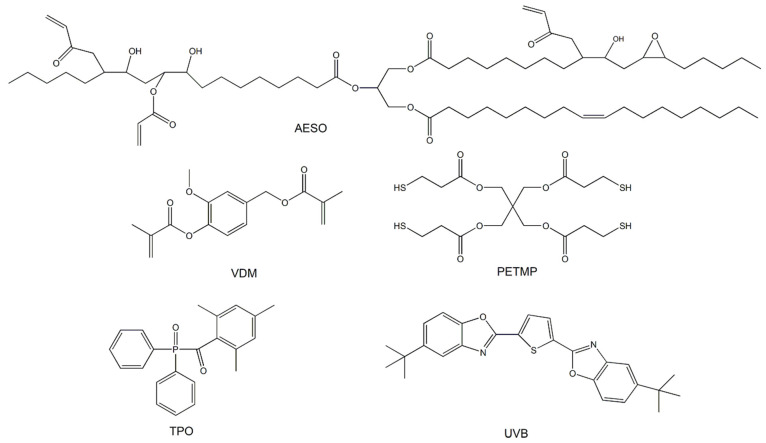
Chemical structures of acrylated epoxidized soybean oil (AESO), vanillin dimethacrylate (VDM), pentaerythritol tetrakis(3-mercaptopropionate) (PETMP), and diphenyl(2,4,6-trimethylbenzoyl) phosphine oxide (TPO).

Acrylated epoxidized soybean oil (AESO, Fluorochem, Glossop, UK), vanillin dimethacrylate (VDM, Specific Polymers, Castries, France), pentaerythritol tetrakis(3-mercaptopropionate) (PETMP, Fluorochem, Glossop, UK), diphenyl(2,4,6-trimethylbenzoyl) phosphine oxide (TPO, Fluorochem, Glossop, UK), and 2,5-bis(5-tert-butyl-2-benzoxazolyl)thiophene (UVB, MPI Chemie, Houten, The Netherlands) (Figure 1) were used as received.

### 2.2. Preparation of Cross-Linked Polymer Specimens

The mixture containing 3 mol of AESO, 1 mol of VDM, 0.25 mol of PETMP, 2.5 wt.% of TPO, and 0.08 wt.% of UVB was stirred with magnetic stirrer at room temperature (25 °C) for 5 min. When the homogeneous mixture was obtained, the resin was poured into a round Teflon mold and cured for 3–5 min in the UV irradiation chamber BS-02 (Opsytec Dr. Grobel, Ettlinger, Germany) with an intensity of 30 mW/cm^2^ and a wavelength range of 280–400 nm. The VS code was assigned to this resin and polymer.

### 2.3. Characterization Techniques

Fourier transformation infrared (FT-IR) spectroscopy spectra were recorded using a Spectrum BX II FT-IR spectrometer (Perkin Elmer, Llantrisant, UK). Reflection was measured during the test. The wavenumber range was 650–4000 cm^−1^.

The Soxhlet extraction was used to determine the yield of the insoluble fraction. A 0.2 g polymer sample was extracted with acetone for 24 h. The insoluble fraction was then dried under vacuum until no changes in weight were observed. The yield of the insoluble fraction was calculated as the weight difference before and after extraction and drying. Three samples of polymer were used to obtain the mean value and standard deviation.

The swelling value of the cross-linked polymer samples was obtained by measuring the mass of the samples swollen in acetone and toluene at room temperature (25 °C). The initial mass of the polymer sample was measured before placing it into the solvent. The change in the mass of the sample was measured every 5 min until no change was obtained. The swelling value was calculated according to the following equation:

(1) $$\alpha =\frac{M-M_{0}}{M_{0}}\cdot 100$$

where α is the swelling value (%); *M* is the mass of the swollen sample (g); *M*_0_ is the initial mass of the sample (g). Three samples of polymer were used to obtain the mean value and standard deviation.

Thermogravimetrical analysis (TGA) was performed on a TGA 4000 apparatus (Perkin Elmer, Llantrisant, UK). A heating rate of 20 °C/min under nitrogen atmosphere (100 mL/min) was chosen. The temperature range of 20–800 °C was used. Aluminium oxide pans were used.

Dynamical mechanical thermal analysis (DMTA) was performed on an MCR302 rheometer (Anton Paar, Graz, Austria). The Peltier-controlled temperature chamber was used. The temperature was increased from −20 °C to 100 °C with a heating rate of 2.0 °C/min. The normal force was set at 5 N during the measurement. In all cases, the shear mode was used with a frequency of 1 Hz and a shear strain of 0.1%. The storage modulus (G’), the loss modulus (G’’), and the loss factor (tan δ) were recorded as a function of temperature. Three polymer samples were used to obtain the mean value and standard deviation.

The mechanical characteristics of the synthesized polymer were determined by the tensile and compression tests. Tensile test was performed on a Testometric M500-50CT testing machine (Testometric Co Ltd., Rochdale, UK) with flat-faced grips at room temperature (20 °C). Bone-shaped polymer specimens with a total length of 70 (±0.0) mm, a shoulder at each end (length of 15 (±0.0) mm, a width of 10 (±0.0) mm), and a gauge section width of 5 (±0.0) mm in between were used. Polymer specimens were printed using the stereolithography technique (SLA) (Section 2.6). The gap between the grips was set to 40 mm and the test was performed at a speed of 5 mm/min until the specimen broke. Young’s modulus, tensile strength, and elongation at break were determined. Five polymer samples were used to obtain the mean value and standard deviation.

The compression test was performed on a Testometric M500-50CT testing machine (Testometric Co Ltd., Rochdale, UK) with HDGG100 grips at room temperature (25 °C). The dimensions of the round tablets used for the test were 15 (±0.10) × 3 (±0.30). The tablets were produced using a Teflon mold. The test was carried out at a speed of 5 mm/min until the sample broke. The compression modulus was determined. Five samples of polymer were used to obtain the mean value and standard deviation.

The biorenewable carbon (BRC) content was calculated according to the following equation:

(2) $$\mathrm{BRC},\;\%=\frac{\mathrm{Bio}\;\mathrm{Sourced}\;\mathrm{Carbon}\;}{\mathrm{Bio}\;\mathrm{Sourced}\;\mathrm{Carbon}+\mathrm{Fossil}\;\mathrm{Carbon}}\;\cdot 100$$

### 2.4. Real-Time Photorheometry

The UV/Vis cure tests of the resin were performed on an MCR302 rheometer (Anton Paar, Graz, Austria) equipped with the plate/plate measuring system. The Peltier-controlled temperature chamber with a glass plate (diameter 38 mm) and a PP15 top plate (diameter 15 mm) was used. The measurement gap was set to 0.1 mm and the samples were irradiated with UV/Vis light in a wavelength range of 250–450 nm through the glass plate using the OmniCure S2000 UV/Vis spot curing system (Lumen Dynamics Group Inc., Mississauga, ON, Canada). The shear mode was used with a frequency of 10 Hz and a shear strain of 1%. The storage modulus (G’), the loss modulus (G’’), and the complex viscosity (η*) were recorded as a function of the irradiation time. The gel point (t_gel_) was calculated as an intersection point of the G’ and G’’ curves. The induction period was measured as the beginning of the increase in G’. The shrinkage was calculated from the reduction in the height of the sample during the photocuring process. The normal force was set to 0 N during the measurement of the sample shrinkage. Five measurements of each resin were used to obtain the mean value and standard deviation.

The crosslinking density was calculated according to the theory of rubber elasticity using the following equation:

(3) $$G^{\prime }=\nu RT$$

where *ν* is the cross-linking density (mol/m^3^); *G’* is the steady-state value of the storage modulus taken from the real-time photorheometry curve after 600 s (Pa); *R* is the universal gas constant (8.314 J/mol K); *T* is the temperature (K) [31]. The experiment was carried out three times to obtain the mean value and standard deviation.

### 2.5. Two-Beam Initiation Threshold Experiment

The 2-BIT experiment allows in situ measurement of non-linear material excitation mechanisms during laser direct writing (LDW) [25]. The 2-BIT experiment was implemented using a Femto second Fiber Laser (FemtoFiber pro NIR, Toptica Photonics AG emitting at 780 nm with pulse duration 150 fs, average output power 500 mW, and repetition rate 80 MHz) (TOPTICA Photonics AG, Munich, Germany). An exact experimental setup of 2-BIT was presented previously [32]. The synthesized polymer was placed between two microscope coverslips (REF VBS638, Biosigma, Cona VE, Italy) separated with a double layer of polyimide film tape. The sample was fixed on piezoelectricstages Nanocube P-611.3S (Physik Instrumente, Karlsruhe, Germany). The 3DPoli software, version 6.22, (Femtika, Vilnius, Lithuania) was used to manage sample positioning. The stages were programmed to make back-forward movements of 75 μm length at a constant velocity of 20 μm/s. The combined laser beams were focused to the sample using the Zeiss Plan-Apochromat oil-immersion, 100×/N.A. = 1.4 objective lens (Carl Zeiss AG, Oberkochen, Germany). At first, the average power *P* (mW) of each beam individually was recorded when the presence of the polymerized features was observed via live imaging and determined as the polymerization threshold for a single beam (*P_single_*). Then, two beams were combined and, while manually controlling the attenuation of both beams with half-waveplates, new polymerization thresholds were recorded (*P_2beam_*). A graph of normalized polymerization thresholds (*P_2beam_/P_single_*) was obtained.

### 2.6. SLA 3D Printing

The SLA 3D printer Phrozen Sonic Mini 4K with a 405 nm LED source was used for polymer sample printing. The printing volume was 134 × 75 × 130 mm, the XY resolution was 35 μm, the layer thickness was 50 μm, and the exposure time was 12 s. After printing, the polymer samples were washed with isopropyl alcohol for 20 min and post-cured under an LED lamp (395 nm, 80 W).

## 3. Results and Discussion

### 3.1. Selection of Resin Composition

**Figure 2:**
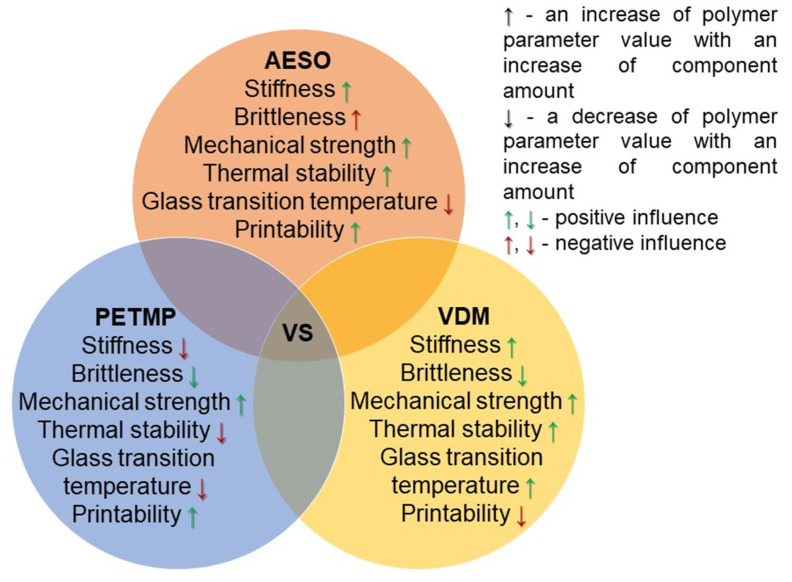
Scheme of the influence of component amounts on some parameters of the polymer VS.

A complex composition of the photocurable resin was made in order to obtain a stable and highly biorenewable carbon content resin suitable for SLA 3D printing of mechanically strong and stiff, but not brittle, polymer objects. The selection of resin components was based on the known features of the compounds and the results of our previous research on this topic. AESO was selected as the main component of the resin due to its biobased origin, the high number of functional groups, the photocuring rate suitable for light-based 3D printing, and the formation of stiff polymer [33]. However, the pure AESO polymer is known to be brittle [9]. VDM was chosen as a biobased aromatic comonomer that improves the mechanical characteristics of the pure AESO polymer [34]. Although pure VDM is not well-suited for SLA 3D printing due to its too-rapid curing and poor stability of the resin during storage [35], in the present study, the aforementioned disadvantages of VDM were not expressed, probably due to its low content in the resin. PETMP, which has four functional groups capable of forming flexible thioether linkages, was added to the mixture of AESO and VDM because it can not only provide flexibility to the rigid polymer network, but also define the shape memory properties of the resulting polymers [36]. The lower-than-stoichiometric amount of thiol groups and acrylic groups allowed for the domination of radical homopolymerization of acrylates and thus obtaining more rigid and mechanically stronger polymers [15]. The flexible aliphatic chains of AESO and PETMP lowered the glass transition temperature of the polymer, while the benzene rings present in VDM and the high amount of AESO functional groups, responsible for the high crosslinking density, led to the high thermal stability of the polymer. Also, the mechanical strength of the polymer is determined by high cross-linking density and aromatic structural fragments. The influence of the amount of each component on some values of the polymer parameters is summarized in Figure 2. Therefore, taking into account all known features of the components, the photocurable resin VS was composed of 3 mol of AESO, 1 mol of VDM, and 0.25 mol of PETMP (acrylic:thiol groups ratio: 2:1), including 2.5 wt.% of the photoinitiator TPO. The resin of such a composition was stable and did not cure during storage for at least six months at a temperature of 4 °C. The calculated biorenewable carbon content of the polymer VS was 76.45%.

### 3.2. Monitoring of Photocuring Kinetics by Real-Time Photorheometry

**Figure 3:**
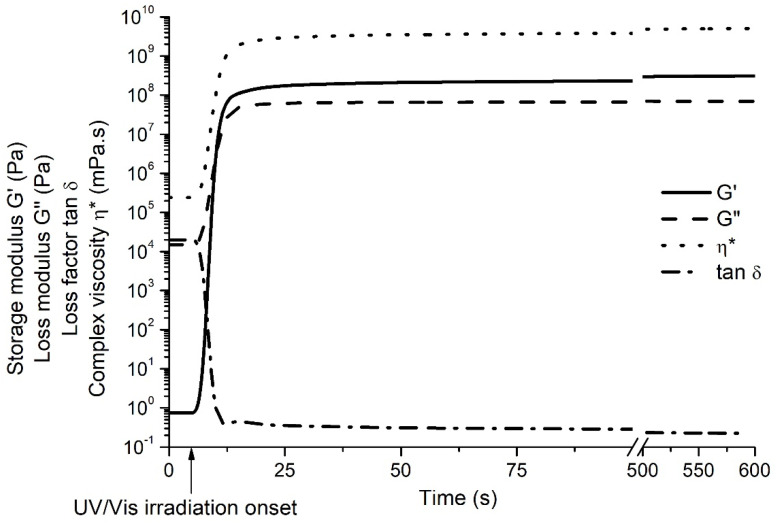
Dependencies of the rheological characteristics of the resin VS on the irradiation time.

The photocuring kinetics of the resin VS was studied by real-time photorheometry. This method was chosen to determine the photocuring rate, the rigidity of the resulting polymer, and also the shrinkage of the sample during photocuring, which are the main factors for the successf of the optical 3D printing process. Figure 3 shows the evolution of the storage modulus G’, loss modulus G”, loss factor tan δ, and complex viscosity η* of the resin VS during UV/Vis irradiation. The cross-linking process began when the values of G’, G”, and η* started to increase. The gel point (t_gel_) (defined as G’ = G”) [37] of the resin was reached after 1.5 s from the onset of UV/Vis irradiation. No induction period was obtained, as the values of G’ and G” started to increase at the same time as UV irradiation started. As the resin irradiation continued, the values of G’, G”, and η* were increasing due to gel aging and then settled into steady state, indicating the end of the cross-linking process. The irradiation was maintained for 600 s. The storage modulus value, which indicates the rigidity of the resulted polymer, reached 317.66 MPa and was more than 20 times higher than those of the other vanillin dimethacrylate- and thiol-based polymers [15]. The shrinkage during photocuring was relatively low and reached 5.5%, which is very important in optical 3D printing technology to produce right-sized objects [38]. Overall, the rheological characteristics make the resin VS a very promising candidate for light-based 3D structuring due to low shrinkage, very high rigidity, and almost instantaneous curing after the start of UV irradiation.

### 3.3. Characterization of the Photocross-Linked Polymer Structure

The chemical structure of the polymer VS was identified by FT-IR spectroscopy. The signals of the C = C group which were present at 1605 cm^−1^ in the FT-IR spectra of AESO and VDM were reduced in their polymer spectra. The signal of the S-H group that was present at 2569 cm^−1^ in the PEMPT spectrum completely disappeared in the polymer VS spectrum, indicating that all S-H groups were consumed in the formation of a polymer network. The FT-IR spectra of AESO, PETMP, VDM, and the cross-linked polymer VS are presented in Figure S1.

**Figure 4:**
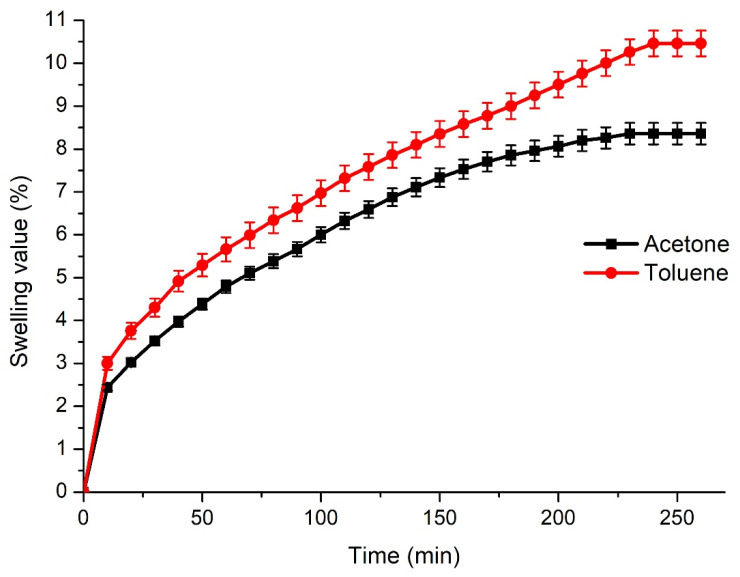
Swelling values of the polymer VS in acetone and toluene.

The Soxhlet extraction was performed, and the crosslinking density was calculated to confirm the crosslinked structure of the polymer. The polymer VS showed a high yield of insoluble fraction (95%) and a high crosslinking density (127,825 ± 206 mol/m^3^), which confirmed that all monomers participated in the formation of the cross-linked structure. The high crosslinking density resulted in low swelling values of the polymer, which were in the range of 8–10% in two solvents of different polarity (Figure 4). The swelling value in toluene was slightly higher than that in acetone. The reason for this is the polymer-solvent interaction, as toluene is a nonpolar solvent and its structure is more similar to the polymer VS in comparison to the polar solvent acetone.

### 3.4. Thermal Properties of Cross-Linked Polymer

**Figure 5:**
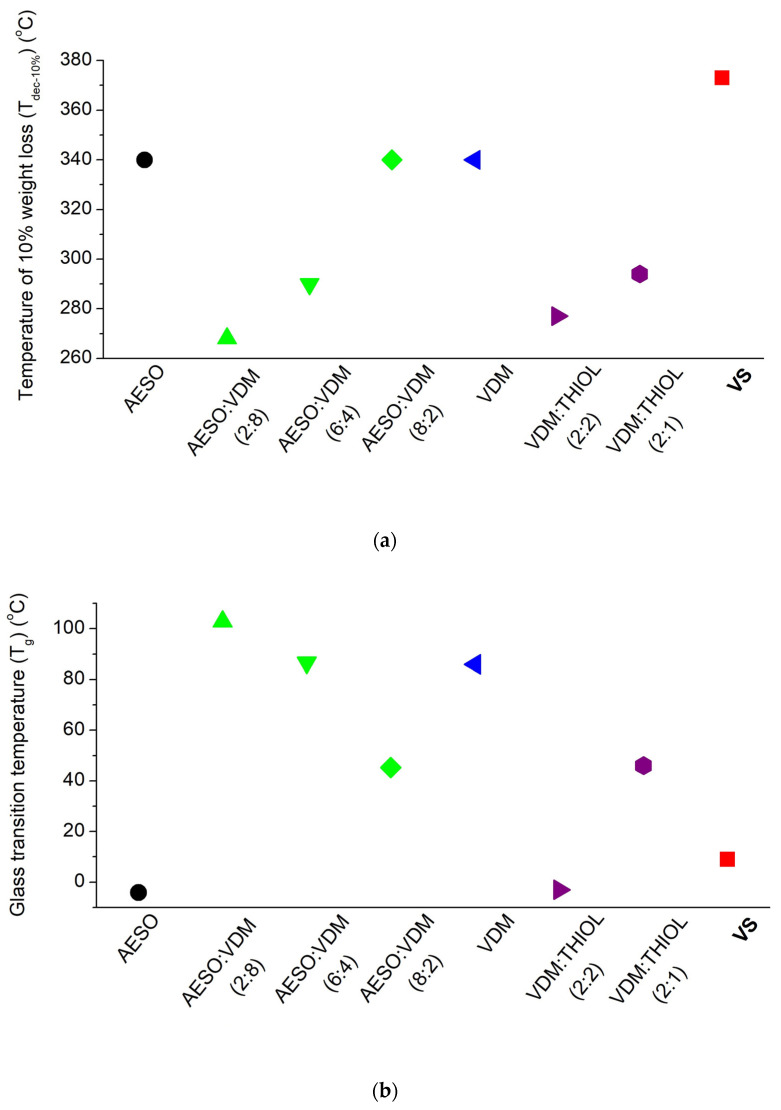
Comparison of the temperature of the 10% weight loss (**a**) and the glass transition temperature (**b**) of the polymer VS and the polymers of AESO [33], VDM [35], AESO and VDM [34], VDM and THIOL [15]. The molar ratio of the monomers is presented in parentheses.

DMTA and TGA were used to study the thermal characteristics of the photocross-linked polymer VS, and the results are presented in Figure S2. These tests were chosen to determine the glass transition and thermal stability of the polymer, which exerted a huge influence on the selection of the application areas of the polymers. The thermal decomposition of the polymer VS occurred in one step. The temperature of 10% weight loss (T_dec -10%_) was 373 °C. The high thermal stability of the polymer was the result of a high yield of the insoluble fraction and a high cross-linking density. The thermal stability of the polymer VS was higher than that of the pure AESO polymer (T_dec -10%_ = 340 °C) [33] and AESO polymers with varying amounts of VDM (T_dec -10%_ = 268–340 °C) [34] (Figure 5). The polymer VS also demonstrated higher thermal stability than the pure VDM polymer and the VDM and thiol copolymer [15,35]. The glass transition temperature (T_g_) of the polymer VS was 9 °C. The low value of T_g_ is due to the high amount of AESO in the polymer. The T_g_ of the pure AESO polymer was reported to be −4.1 °C and the addition of VDM could increase it to 102.9 °C [34] (Figure 4). In this study, the addition of VDM increased T_g_; however, the addition of PETMP contributed to a decrease, which resulted in a T_g_ value of the polymer VS which was close to room temperature, making it both rigid and flexible at this temperature.

### 3.5. Mechanical Characteristics of Cross-Linked Polymers

**Figure 6:**
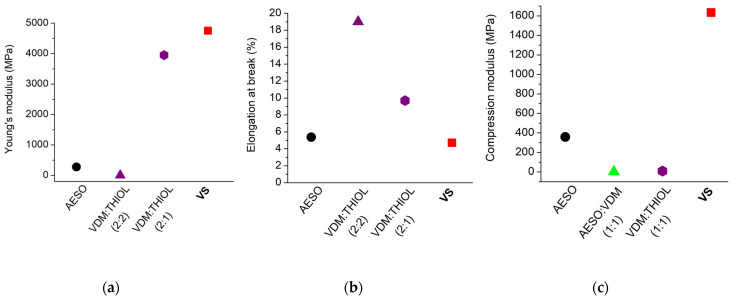
Comparison of Young’s modulus (**a**), elongation at break (**b**), and compression modulus (**c**) of the polymer VS and the polymers of AESO [33], AESO and VDM [40], VDM and THIOL [15,39]. The molar ratio of the monomers is presented in parentheses.

The most commonly-used tensile and compression tests were performed to determine the mechanical characteristics of the polymer VS for the evaluation of its mechanical strength, stiffness, and brittleness. The results are presented in Table S1 and Figure S3. The high value of Young’s modulus and the low value of elongation at break show that the polymer VS is a rigid and stiff material (Table S1). Compared to other vanillin dimethacrylate- and thiol-based polymer VDM/THIOL, the polymer VS is more rigid and less flexible, as the other polymer based on VDM reached a lower Young’s modulus value (3952.3 MPa) and a higher elongation at break value (9.7%) [15] (Figure 6). The polymer VS is also mechanically stronger than the pure AESO polymer, which demonstrated a lower value of Young’s modulus. The compression test confirmed that the polymer VS was a less brittle material. The compression modulus reached 1633.72 MPa and was much higher than that of the other vanillin dimethacrylate- and thiol-based polymer VDM/THIOL [39], the pure AESO polymer, and the polymer AESO/VDM [40]. These results show that the polymer VS can be used in applications that require mechanically-strong polymers.

### 3.6. SLA 3D Printing

**Figure 7:**
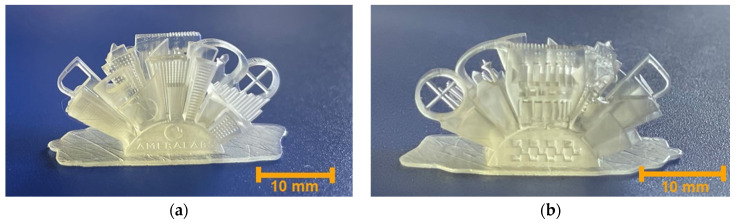
Images of SLA 3D printed polymer structures. The front side is on the left (**a**), and the back side is on the right (**b**).

The composed resin VS was successfully applied in the SLA 3D printing technology. Complex shape structures were printed with high accuracy and a smooth surface finish, confirming the suitability of this biobased resin for the SLA 3D printing technology. The images of the 3D printed ‘Ameralabs Town’ are presented in Figure 7.

The main limitation of the application of the VS resin in SLA 3D printing was the high viscosity, which resulted in a relatively slow 3D printing process at room temperature due to the necessary long wait period to recoat a new layer [41]. However, a high viscosity of the photocurable resin is preferred in laser direct writing technology, as it limits the mobility of oxygen and radicals that terminate the propagation, resulting in a decrease in polymerization thresholds and an increased dynamic range (difference between polymerization and optical damage thresholds) [42]. Moreover, the results of the 2-BIT experiment showed that high viscosity can be overcome by using a 3D printer with a heating function, as increasing the temperature also increases the rate of photopolymerization.

### 3.7. Determination of the Polymerization Threshold Using the 2-BIT Method

**Figure 8:**
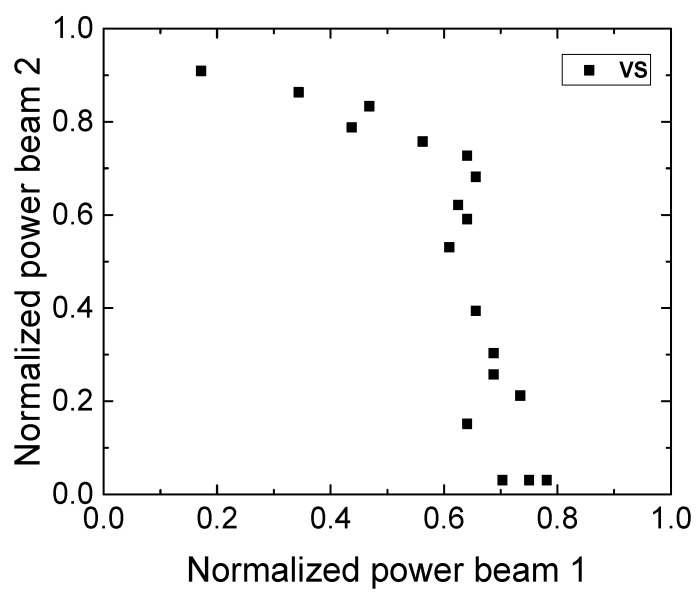
2-BIT data for the polymer VS prepared at room temperature.

The results of the 2-BIT experiment are provided in Figure 8. The normalized power of beam 2 vs the normalized power of beam 1 is depicted. Normally, it is expected that, when decreasing the power of beam 2, beam 1 should be proportionally increased to observe the polymerized features. Eventually, when beam 2 is close to the value of 0, beam 1 should reach the value of 1, which is determined as the polymerization threshold when a single beam was used. In this study, this value was *P_single_* = 6.4 mW for beam 1 and *P_single_* = 6.6 mW for beam 2. This did not allow for direct assessment of *n_eff_* in the used photocurable resin but provided some interesting and useful data while varying sample preheating conditions.

In this case, the power of beam 1 converged to 0.8 when beam 2 was close to 0. During the duration of the experiment of approximately 1 h, a noticeable decrease of 33% was determined when *P_single_* was reduced from 6.4–6.6 mW to 4.3–4.4 mW. This prohibits the determination of effective order or nonlinearity due to the variable photopolymerization threshold, which might be attributed to thermal effects under ambient conditions. To confirm that the threshold was changing specifically due to thermal effects (Brownian motion of molecules that increase photopolymerization efficiency), the resin was placed on the hot plate for 2 h at 65 °C. In this case, *P_single_* was recorded to be 0.8 mW and 2.8 mW for beam 1 and beam 2, respectively, resulting in a decrease which was significant more than 50% in used laser power. This is an important material feature for reducing the required laser energy consumption in the industrial applications context. A liquid form of the TPO (TPO-L) photoinitiator was used in the resist composed of tris (2-hydroxy ethyl) isocyanurate triacrylate and dipentaerythritol pentaacrylate for the 2-BIT experiment by the authors of reference [24]. The resist was excited with a laser generating 800 nm wavelengths and *n_eff_* was determined to be equal to 2, resulting in the absorption of two photons at 400 nm. As both TPO and TPO-L have identical absorption spectra, a similar result was expected in this work.

## 4. Conclusions

A novel stable high biorenewable carbon content photopolymerizable resin was developed from acrylated epoxidized soybean oil, vanillin dimethacrylate, and pentaerythritol tetrakis(3-mercaptopropionate)-based polymer and was successfully applied in SLA 3D printing technology. The calculated biorenewable carbon content of the synthesized polymer was 76.45%. The resin demonstrated rapid photocuring without an induction period and reached a rigidity of 317.66 MPa, which was more than 20 times higher than that of the other vanillin-based polymers. The polymer with a high crosslinking density (127,825 ± 206 mol/m^3^) showed excellent mechanical properties (Young’s modulus was 4753 MPa and the compression modulus was 1634 MPa) and great thermal stability (the temperature of 10% weight loss was 373 °C). The developed stable dual-cure photopolymerizable system is suitable for light-based 3D structuring of objects with complex shapes. Based on the 2-BIT experiment, it was determined that the polymerization threshold of the investigated polymer can be controlled by sample temperature and was reduced to the lower values by a f mW (≈50% of the total laser power > 6 mW needed to induce photocuring). This is a great advantage, as it opens up the employment of dual-cure resins for low-power laser systems in LDW, making the footprint of the workstations cheaper, faster, and more reliable.

## Data Availability

Not applicable.

## Supplementary Materials

The following supporting information can be downloaded at: https://www.mdpi.com/article/10.3390/polym14245361/s1, Figure S1: FT-IR spectra of AESO, PETMP, VDM, and the polymer VS; Figure S2: Thermogravimetric curve (a) and DMTA thermogram (b) of the polymer VS; Figure S3: Tensile stress-strain curves of cross-linked polymer (samples 1–5). Table S1: Mechanical characteristics of the cross-linked polymers [15,25,30,31].
