# Supplementary material for: Functionalized Soybean Oil- and Vanillin-Based Dual Cure Photopolymerizable System for Light-Based 3D Structuring
Source: Polymers (Basel). 2022 Dec 8;14(24):5361. doi: 10.3390/polym14245361 (PMC9781298; doi:10.3390/polym14245361)
Supplement: Supplementary file 1 [file polymers-14-05361-s001.zip › polymers-2074570-supplementary.pdf]

polymers

MDPI

Article

Functionalized Soybean Oil- and Vanillin-Based Dual-Cure Photopolymerizable System for Light-Based 3D Structuring

Vilte Sereikaite[1], Aukse Navaruckiene[1], Justinas Jaras[1], Edvinas Skliutas[2], Dimitra Ladika[3], David Gray[3], Mangirdas Malinauskas[2], Vaidas Talacka[4] and Jolita Ostrauskaite[1,*]

[1] Department of Polymer Chemistry and Technology, Kaunas University of Technology, Radvilenu Rd. 19, LT-50254 Kaunas, Lithuania
[2] Laser Research Center, Faculty of Physics, Vilnius University, Sauletekis Ave. 10, LT-10223 Vilnius, Lithuania
[3] Institute of Electronic Structure and Laser, Foundation for Research and Technology – Hellas, 70013 Heraklion, Greece
[4] AmeraLabs, Kęstučio Str. 6A, LT-44320 Kaunas, Lithuania
[*] Correspondence: jolita.ostrauskaite@ktu.lt (J.O.); Tel.: +370-37-300192

Citation: Sereikaite, V.; Navaruckiene, A.; Jaras, J.; Skliutas, E.; Ladika, D.; Gray, D.; Malinauskas, M.; Talacka, V.; Ostrauskaite, J. Functionalized Soybean Oil- and Vanillin-Based Dual Cure Photopolymerizable System for Light-Based 3D Structuring. *Polymers* **2022**, *14*, 5361. https://doi.org/10.3390/polym14245361

Academic Editor: Arpan Biswas

Received: 18 November 2022
Accepted: 6 December 2022
Published: 8 December 2022

Publisher's Note: MDPI stays neutral with regard to jurisdictional claims in published maps and institutional affiliations.

Copyright: © 2022 by the authors. Submitted for possible open access publication under the terms and conditions of the Creative Commons Attribution (CC BY) license (https://creativecommons.org/licenses/by/4.0/).

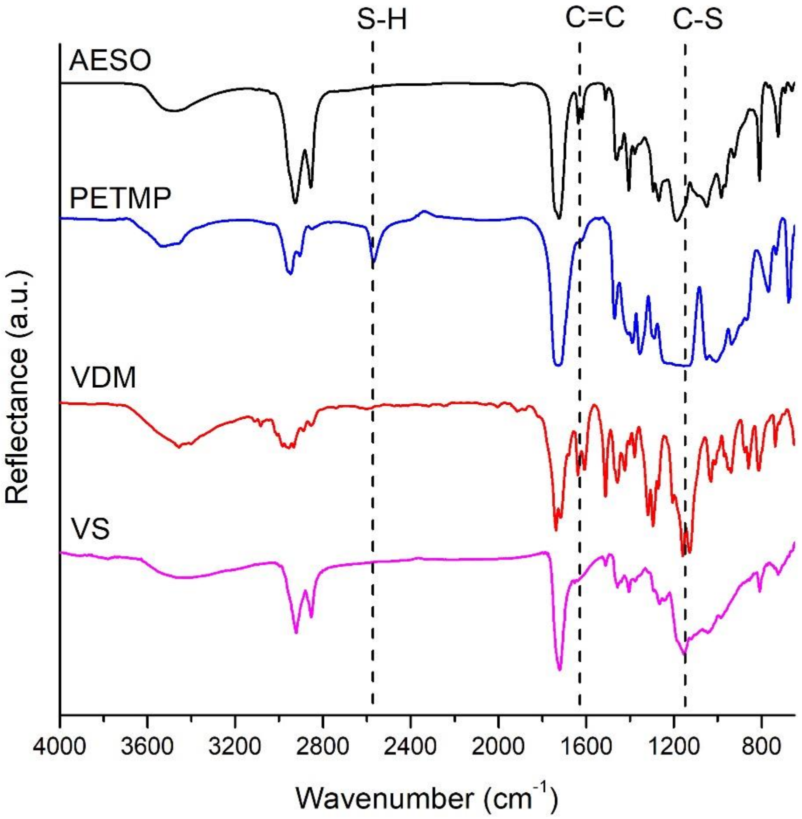

Figure S1. FT-IR spectra of AESO, PETMP, VDM, and the polymer **VS**

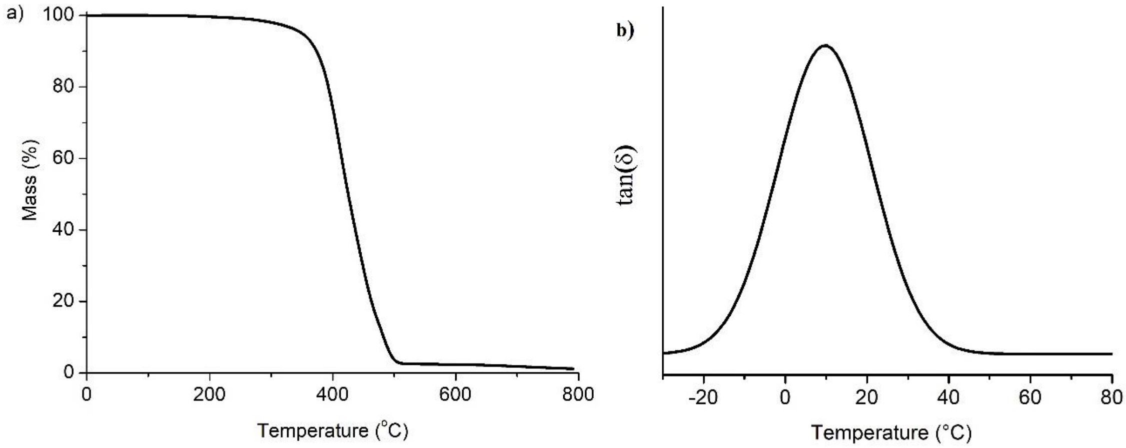

Figure S2. Thermogravimetric curve (a) and DMTA thermogram (b) of the polymer **VS**.

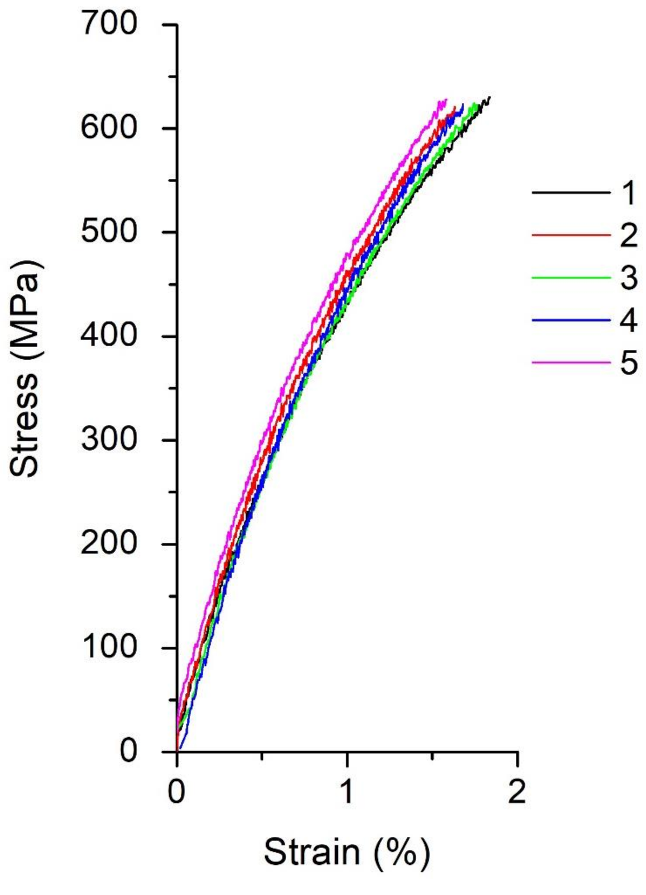

Figure S3. Tensile stress-strain curves of cross-linked polymer (samples 1-5).

Table S1. Mechanical characteristics of the cross-linked polymers

Polymer	Young's modulus, MPa	Tensile strength, MPa	Elongation at break, %	Compression modulus, MPa
VS	4753.10 ± 175.20	88.54 ± 4.26	4.72 ± 0.49	1633.72 ± 78.74
**VDM/THIOL (2:2)** [15]	1.4 ± 0.2	0.02 ± 0.0	19.0 ± 3.2	-*
**VDM/THIOL (2:1)** [15]	3952.3 ± 52.3	177.9 ± 11.6	9.7 ± 0.3	-
**AESO** [25]	279 ± 3	5.39 ± 1.01	1.6 ± 0.36	359 ± 20
**AESO/VDM** (1:1) [31]	-	-	-	9.9 ± 0.2
**VDM/THIOL** (1:1) [30]	-	-	-	0.70 ± 0.02

* - not reported
